# Element mobility during basalt-water-CO_2_ interaction: observations in natural systems vs. laboratory experiments and implication for carbon storage

**DOI:** 10.1186/s12932-024-00087-7

**Published:** 2024-05-16

**Authors:** Pierangelo Romano, Lorenzo Brusca, Marcello Liotta

**Affiliations:** Istituto Nazionale di Geofisica e Vulcanologia Sezione di Palermo, Via Ugo la Malfa 143, Palermo, 90100 Italy

**Keywords:** Element mobility in groundwaters, Rock-water-CO_2_ interaction processes, CO_2_ storage in mafic rocks

## Abstract

Today, carbon dioxide removal from the atmosphere is the most ambitious challenge to mitigate climate changes. Basalt rocks are abundant on the Earth’s surface (≈ 10%) and very abundant in the ocean floors and subaerial environments. Glassy matrix and minerals constituting these rocks contain metals (Ca^2+^, Mg^2+^, Fe^2+^) that can react with carbonic acid to form metal carbonates (CaCO_3_, MgO_3_ and FeCO3). Here, we present a data compilation of the chemical composition of waters circulating in basalt aquifers worldwide and the results of simple basalt-water-CO_2_ experiments. Induced or naturally occurring weathering of basalts rocks release elements in waters and elemental concentration is closely dependent on water CO_2_ concentration (and hence on water pH). We also performed two series of experiments where basaltic rock powder interacts with CO_2_-charged waters for one month at room temperature. Laboratory experiments evidenced that in the first stages of water-rock interaction, the high content of CO_2_ dissolved in water accelerates the basalt weathering process, releasing in the water not only elements that can form carbonate minerals but also other elements, which depending on their concentration can be essential or toxic for life. Relative mobility of elements such as Fe and Al, together with rare earth elements, increases at low pH conditions, while it decreases notably at neutral pH conditions. The comparison between experimental findings and natural evidence allowed to better understand the geochemical processes in basaltic aquifers hosted in active and inactive volcanic systems and to discuss these findings in light of the potential environmental impact of CO_2_ storage in mafic and ultramafic rocks.

## Introduction

Carbon dioxide is one of the minor constituents of the Earth’s atmosphere that consists of nitrogen (N_2_ = 78%) and oxygen (O_2_ = 21%), the remaining 1% including argon and the carbon species (CO_2_, CO) in concentrations of about 0.04% and other minor gases (< 0.001%). However, the interaction between water-CO_2_ and mafic rocks has important geochemical relevance, such as the control in the carbon global cycle on geological time scales. The carbon released from the crust and mantle is balanced with the weathering of Ca-Mg- silicate and the formation of carbonates [1–3]. In addition, the presence of carbon in the atmosphere is the basis of life as we know it today.

Since the beginning of the Industrial Revolution, the cumulative release of anthropogenic carbon has grown exponentially [4] and the emissions in the atmosphere are estimated around 40 GtCO_2_/yr [5]. Ocean, biosphere and rock weathering, as part of the natural carbon cycle, naturally remove half carbon that enters each year in the atmosphere. The reduction of carbon dioxide (CO_2_) emissions into the atmosphere is hence one of the greatest challenges of this century, requiring engagement at different society levels. Several options for CO_2_ removal from the atmosphere have been proposed with the final aim of removing approximately 10 Gt/yr of CO_2_ by 2050 ([6] and reference therein). Carbon mineralization is one of the methods widely investigated in the laboratory and pilot-scale projects [7–16], it is a non-toxic and long-term method of storing CO_2_ in a solid form. Carbon mineralization is usually settled by water, when CO_2_-saturated waters are injected into large volumes of mafic and ultramafic rocks. In this way, carbon dioxide is fixed in minerals during the interaction with divalent cations (Ca^2+^, Mg^2+^, Fe^2+^ etc.) to form carbonate minerals such as CaCO_3_, Ca_0.5_Mg_0.5_CO_3_, MgCO_3_, FeCO_3_. In natural systems the rainwater that infiltrates the rocks and contributes to the aquifers usually has a pH ∼ 5 and a variable content of cations and anions that may influence the weathering processes of minerals and rocks. The composition of rainwater is itself influenced by several factors such as the seasonality, the environmental conditions (wind, temperature, pressure), the presence of volcanic plumes, and natural and anthropic dust particles. Hence, groundwater composition is the result of several processes, including the ongoing weathering process, the composition of rainwater and the effect of gases eventually released from volcanic plumes or via soil diffuse degassing. Several authors suggest that in active volcanic systems most of the CO_2_ is lost through non-eruptive degassing and solubilized in groundwater [17–19] so that the CO_2_ is added to the aquifer. Element mobility in volcanic groundwaters has been studied in several volcanic systems [17, 19–27] and a large number of experimental studies [7–9, 28] have been done to understand the dissolution rates and the reaction path during water-CO_2_ rock interaction. The element mobility is studied by computing the relative mobility (RM) value for each element, expressed as the water-rock element concentration ratio normalized to a specific element [17, 29] or studying the mineralogy of the rock weathering profile [21, 30, 31]. Several works focused on the influence of pH on the dissolution rate of simple compounds in the crystalline (i.e. feldspar, plagioclase or olivine) or amorphous form [32 reference therein]. Temperature, pH, and Eh can influence the behaviour of trace elements during the weathering process. Depending on their concentration, these elements can be essential or toxic for the biota [9, 19, 33, 34]. In this work, we first present a review of groundwater composition circulating in basaltic aquifers worldwide, comparing data on active and inactive volcanic systems. As suggested by Flaathen et al. [19], the study of natural systems is a method to assess risk and potential of CO_2_ sequestration in basaltic rocks. We then discuss the data obtained from simple rock-water-CO_2_ interaction experiments focusing our attention on the solute chemistry. Previous experimental works were aimed at studying the water chemistry and the alteration mineralogy as a function of time at different temperature conditions and with different amounts of CO_2_ dissolved in water. In our experiments, we sought to approximate *quasi*-equilibrium conditions of the natural systems by performing experiments with fixed amounts of rock and water and an excess of CO_2_. We compare natural water composition worldwide, experimental results, and the knowledge of Etna volcano groundwater system, which undergoes inputs of magmatic gases [35]. The final aim of this paper is to provide further insights into the elements behaviour in water circulating in basaltic aquifers and discuss the consequences of injecting CO_2_ in mafic and ultramafic rocks from a geochemical point of view.

## Materials and methods

### Data compilation

Data on the water compositions circulating in several basalt aquifers worldwide were collected. The data belong to scientific papers published in the last 30 years, relevant to studies on water compositions and hydrogeology of the aquifers. From the literature (26 articles, supplementary materials), we selected only the water compositions sampled in wells and springs, excluding river and rain water, these latter being unsuitable for our objectives. In Table 1, we provide information on the localities of the selected aquifers, distinguishing information relative to the state of the volcanic system to which the aquifer belongs (active or inactive), the range of alkalinity and pH. The parameters considered for our work are water alkalinity, pH and major and minor element composition as well; these data will be described in the results section and discussed later in the text.

**Table 1:** Localities of Basaltic aquifers

Reference	Localities	Sample site	Temperature (°C) range	pH range
Louvat & Allegre, 1997	La Reunion	thermomineral spring	48-29.5	6.2–6.7
Louvat & Allegre, 1998	Sao Miguel	thermal spring	26	6.5
Benedetti et al., 1994	Parana Traps	spring and drill hole	-	6.6–8.7
Stefansson et al., 2001	Islanda Laxa ´ in Kjo ´ s catchment area	spring and drill hole	18-24.8	7.3–8.4
Riotte et al., 2003	Camerun- Mount Cameroon	spring	16.3–23.5	6.4–8.1
Liotta et al., 2016	Etna	spring and groundwater	10.9–19.4	6.0-7.5
Gastmans et al., 2016	Serra Geral Aquifer, São Paulo state (Brazil)	groundwater	23.5–34.7	5.7–9.9
Kamble et al., 2013	Deccan Volcanic Province (southern margin)	groundwater and well	-	7.2–8.3
Fenta et al., 2020	Northwest Ethiopia	Borehole, Shallow well, Spring	-	6.6–8.1
Tweed et al., 2006/2004	Dandenong Ranges, southeast Australia	groundwaters and boreholes	-	6.1–8.6
Raiber et al., 2009	basalt plains of southeastern Australia	boreholes	-	-
Dafny et al., 2006	Golan Heights (Israel)	groundwater, well and spring	15-23.1	6.5–8.4
Prada et al., 2005	Madeira	spring and tunnel in perched acquifer	10.1–19.7	6.1–8.4
Duckett et al., 2019/2020	Columbia River (pay Grande Ronda Well	well	12.1–17.2	7.4-8.0
Liu et al., 2015	Columbia River Basalts	well	10.9–28.6	7.7–8.8
Siebert et al., 2014	Yarmuck Gorge & Golan Heights	well	19.1–24.3	8.3–9.07
Koh et al., 2009	Jeju Island, South Korea	spring and well	8.6–20.3	6.6–8.2
Flaathen et al. 2009	Mount Hekla, Iceland	groundwater, well, spring, cold spring	1.9-8.0	7.2–9.2
Cruz et al., 2009	Cruz et al., 2009	groundwater, well, spring, cold spring	14.3–18.3	7.01–7.63
Cordeiro et al., 2012	Sao Miguel, Azores	groundwater, well and spring	12.1–17.7	6.5–7.6
Ludwig et al., 2012	Mt. Wasserkuppe, Germany	groundwater and spring	6.1–11.8	6.6–9.7

### Description of the experimental setup

Rock water-CO_2_ interaction experiments were carried out using a rock sample (Table 2) erupted by Etna volcano in 2011 during one of the 18 lava fountains ([36] and reference therein). Following the T.A.S classification (Total Alkali versus Silica), the rock sample has a K-trachybasalt composition with a total phenocrysts abundance ranging from 15 to 30 vol%. Plagioclase dominates the mineral phases with respect to the mafic minerals (clinopyroxene + olivine + oxide) and the groundmass is generally glassy to cryptocrystalline. Analyses of major and trace elements have been obtained by ICP-OES and ICP-MS at the Centre de Recherches Petrographiques et Geochimiques (CNRS-CRPG) in Nancy (France). Relative uncertainty (1σ) is < 2% for SiO_2_ and Al_2_O_3_, < 2% for Fe_2_O_3_, MgO, CaO, Na_2_O, K_2_O, < 5% for MnO, and TiO_2_ and 5–10% for P_2_O_5_, and < 5% for all trace elements except U (< 8%). Major and trace element composition is reported in Table 2. The rock samples were finely crushed to a grain size of 10–30 μm and then loaded in 120 ml polypropylene bottles, together with distilled water and CO_2_. Water content loaded in the bottles was 100 ml and rock-water ratios (Table 3) were fixed by changing the amount of rock powder loaded in the bottle. In Run1 (R1), the rock-water ratio varies from 4.79*10^− 4^ to 1.91*10^− 3^, in Run 2 (R2) from 5.92*10^− 2^ to 4.66*10^− 1^, the amount of rock powder was 0.05 g in Run 1–1, while in Run 1–2 and Run 1–3 we loaded respectively 0.1 and 0.2 g. Run 2 differs for the amount of rock powder loaded, in Run 2 − 1 ∼ 5 g were loaded, namely two orders of magnitude greater with respect to R1-1, in R2-2 20 g and in R2-3 46.7 g (Table 3). CO_2_ was loaded in the bottle with rock and water through a syringe and a modified cap of the bottles. In order to assure the dissolution of CO_2_ in the water, the syringe was connected to the bottle with a silicon tube and the syring loaded of gas was kept under pressure for 24 h applying a constant force to the syringe piston. After 24 h the syringe presented amount of undissolved gas, this confirmed the dissolution and excess of CO_2_ within the polypropylene bottle. Once loaded, the polypropylene bottles were placed in a tilting table for one month in order to ensure a complete and constant mixing between solids and fluids during the experiments. At the end of the experiment, four aliquots of water were taken, filtered with 0.22 μm filter, and placed in plastic containers (Falcon®) for the following analyses; three of the four water aliquots were also acidified with HNO_3_.

**Table 2:** Major and trace elements of the basaltic rock used in the experiments

		CSE190711A	SG-01
SiO2	%	46.36	13.21
TiO2	%	1.732	0.001
Al2O3	%	16.858	0.07
Fe2O3	%	11.515	56.56
MnO	%	0.1847	0.242
MgO	%	5.529	0.21
CaO	%	10.508	2.39
Na2O	%	3.922	0.05
K2O	%	1.997	0.04
P2O5	%	0.59	0.78
F	%	0.11	
S (t)	%	0.03	
Cl	ppm	610	
Total	%	99.15	98.61
PF	%	-0.04	25.05
Li	µg/g	7.9	
Ag	µg/g	< 0.10	
B	µg/g	9.4	
As	µg/g	2.0306	10
Ba	µg/g	619.4225	145
Be	µg/g	1.7581	3
Bi	µg/g	0.0464	< 0.1
Cd	µg/g	0.2995	
Co	µg/g	43.1951	27
Cr	µg/g	26.452	< 20
Cs	µg/g	0.8071	< 0.1
Cu	µg/g	131.0296	< 10
Ga	µg/g	22.5524	< 1
Ge	µg/g	1.5724	< 0.5
Hf	µg/g	4.3557	< 0.1
In	µg/g	0.2381	< 0.1
Mo	µg/g	2.8433	5
Nb	µg/g	42.3296	< 0.2
Ni	µg/g	24.5427	< 20
Pb	µg/g	4.8252	< 5
Rb	µg/g	48.9255	< 1
Sb	µg/g	0.1358	1
Sc	µg/g	28.17	< 1
Sn	µg/g	2.0005	< 1
Sr	µg/g	1188.5235	391
Ta	µg/g	2.432	0.01
Th	µg/g	7.2036	< 0.05
U	µg/g	2.0555	0.2
V	µg/g	296.2804	25
W	µg/g	18.3455	< 0.5
Y	µg/g	26.0144	2.2
Zn	µg/g	108.2607	30
Zr	µg/g	198.0781	6
La	µg/g	54.9639	0.64
Ce	µg/g	106.3964	0.8
Pr	µg/g	11.9872	0.13
Nd	µg/g	46.22	0.63
Sm	µg/g	8.8331	0.15
Eu	µg/g	2.642	0.026
Gd	µg/g	7.2232	0.16
Tb	µg/g	0.9852	0.02
Dy	µg/g	5.4003	0.11
Ho	µg/g	1.0121	0.03
Er	µg/g	2.4798	0.12
Tm	µg/g	0.3474	0.018
Yb	µg/g	2.0832	0.12
Lu	µg/g	0.3109	0.018

**Table 3:** Experimental setup and results

ID sample	Water (ml)	Rock (g)	Rock-water ratio	pH	Alkalinity meq/l	H^+^	C (mol)	CO_2_gas (mol)
CSE R-1-1	106.57	0.051	4.79E-04	5.05	0.11	8.913E-06	1.17E-05	4.10E-03
CSE R-1-2	107.10	0.103	9.62E-04	5.01	0.152	9.772E-06	1.63E-05	4.10E-03
CSE R-1-3	104.02	0.198	1.91E-03	5.13	0.183	7.413E-06	1.90E-05	4.10E-03
CSE R2-1	100.14	5.7	5.69E-02	5.98	3.15	1.047E-06	3.15E-04	4.10E-03
CSE R2-2	101.26	20.93	2.07E-01	6.39	9.6	4.074E-07	9.72E-04	4.10E-03
CSE R2-3	100.11	46.70	4.66E-01	6.98	13.965	1.047E-07	1.40E-03	4.10E-03

### Water analysis

Water composition was determined at the Istituto Nazionale di Geofisica e Vulcanologia (Sezione di Palermo). The pH measurements were performed using a Mettler Toledo electrode model DGi 115SC connected to a compact titrator model G20. Major element composition anions (F^−^, Cl^−^, NO_3_^−^, SO_4_^−−^) and cations (Na^+^, K^+^, Mg^++^, Ca^++^) were determined by using an ion chromatography system Thermo Scientific Dionex ICS-5000 in suppressed mode and equipped with an anion column (AS19) and a precolumn (AG19A) working under continuous flow of the carbonate–bicarbonate eluent, and a cation column (CS16) and precolumn (CG16) that works under continuous flow of methanesulfonic acid with eluent regeneration. Precision and accuracy of the method are described in Prano and Liotta [37]). Trace elements were analysed by inductively coupled plasma mass spectrometry (ICP-MS, Agilent 7800) and inductively coupled plasma optical emission spectroscopy (ICP-OES, Horiba Ultima 2). Due to the low elements concentration, major elements of the experimental set R1 were also analysed by ICP-OES.

## Results

### Characteristics of waters in basaltic aquifers worldwide

**Fig. 1:**
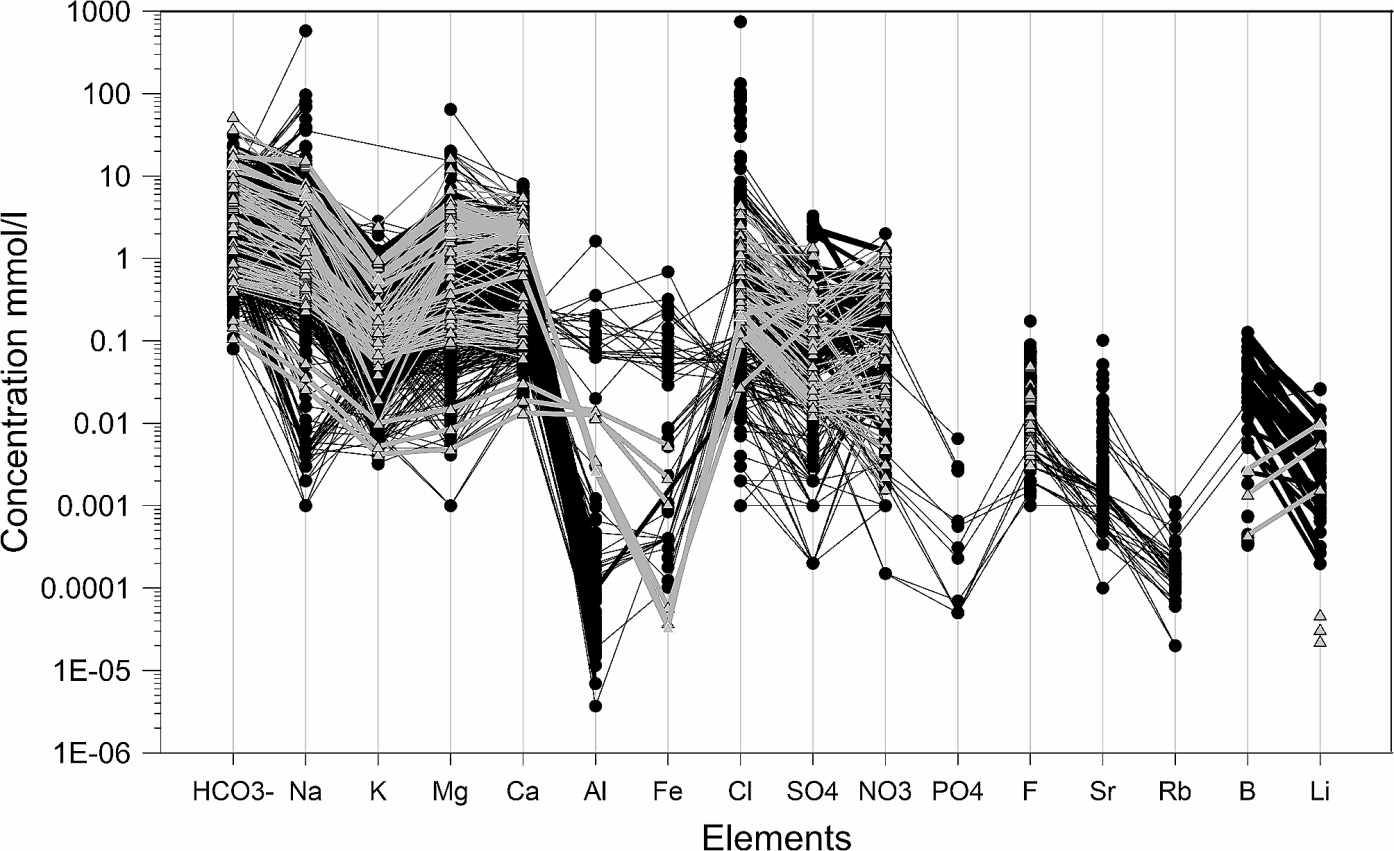
Concentration of major and trace elements in basaltic aquifers worldwide

Water composition of basaltic aquifers worldwide is shown in Fig. 1. pH and water temperature conditions, depending on the location, are in the range 6–8 and 5–47 °C respectively. Alkalinity values range from 0.1 to 100 meq/l with most of the data in the range 1–10 meq/l. Most of the water compositions taken into account present Na content in the range 0.001 to 100 mmol/l, K in the range 0.001 and 1 mmol/l, while Mg and Ca concentrations are in the range 0.001–10 mmol/l and 0.01–10 mmol/l respectively. Anion concentration ranges mostly between 0.1 and 1 mmol/l (Cl and SO4^−−^). As regards other minor elements in natural waters, the data set is complete only for a few elements such as Al, Fe, Rb Sr, B, Li; which are available only for a few localities. Looking in detail at the dataset and Fig. 1, Al concentration ranges between 1*10^− 5^ and 0.001 mmol/l, while Fe ranges between 0.0001 and 0.001 mmol/l. Only water samples of a specific locality show Al and Fe content significantly higher with respect to the rest of the dataset, ranging both between ∼ 0.01 and 1 mmol/l. Regarding Sr, B and Li, the concentration range is between ∼ 0.0001 and 0.1 (Li 0.0001-0.01), while Rb concentration varies between 0.0001 and 0.001 mmol/l.

### Experimental results

**Fig. 2:**
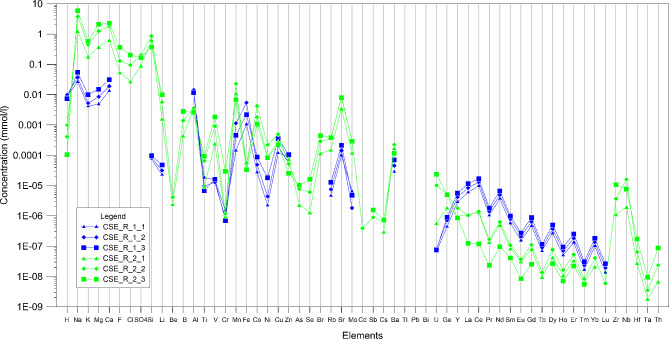
Concentration of major and trace elements in the water solutions obtained after the water-CO2 basalt interaction experiments

We performed experiments in order to study the composition of water after a long (1 month) rock-water-CO_2_ interaction process with the final aim of studying the element mobility. The experiments were carried out by loading an excess of CO_2_ and waiting for equilibrium dissolution of the gas in the water. Although we were not able to verify the water pH at the beginning of the experiments, it is possible to assume an initial pH value considering at least 1 bar of CO_2_ in equilibrium with water at a temperature of 25 °C. Hence, the pH can be considered at least equal or lower than 5. At the end of the experiments, waters of experiments R-1-1 to R-1-3 have pH in the range 5.01 to 5.13 and alkalinity in the range 0.11 to 0.18 meq/l. In the experimental set R2, we observed a variation in pH in the range 5.98 to 6.98, while alkalinity varies between 3.00 and 13.96 meq/l. Major and trace element concentration of the experimental waters is reported in Table S1 and plotted in Fig. 2. Keeping in mind the difference in the rock water ratio described in the previous section, waters of experimental sets R1 have a concentration of major elements in the order of hundredths and thousandths of mmo/l, while trace elements are in general two or three orders of magnitude lower. In the experimental set R2, major elements have a concentration in the range of tenth to unit of mmol/l; as regards the trace elements, their concentrations vary between 0.01 and 1*10^− 6^ mmol/l (Fig. 2); in both experimental sets, rare earth elements have a concentration in the range 1*10^− 8^ − 1*10^− 5^. Si, Al, Ti, Fe, Mn, represent together with Na, K, Mg, Ca the major constituent of the K-trachybasalts rock sample and have a concentration in the range 0.01 to 1 *10^− 5^ mmol/l. Anion concentrations (S, F, Cl) have been determined only for the experiments set R2. Sulphur (expressed as sulphate ion) concentration varies between 0.091 and 0.167 mmol/l, while chlorine and fluorine are in the range 0.0275–0.203 mmol/l and 0.054–0.36 mmol/l, respectively.

## Discussion

### Water composition in basaltic aquifers worldwide

**Fig. 3:**
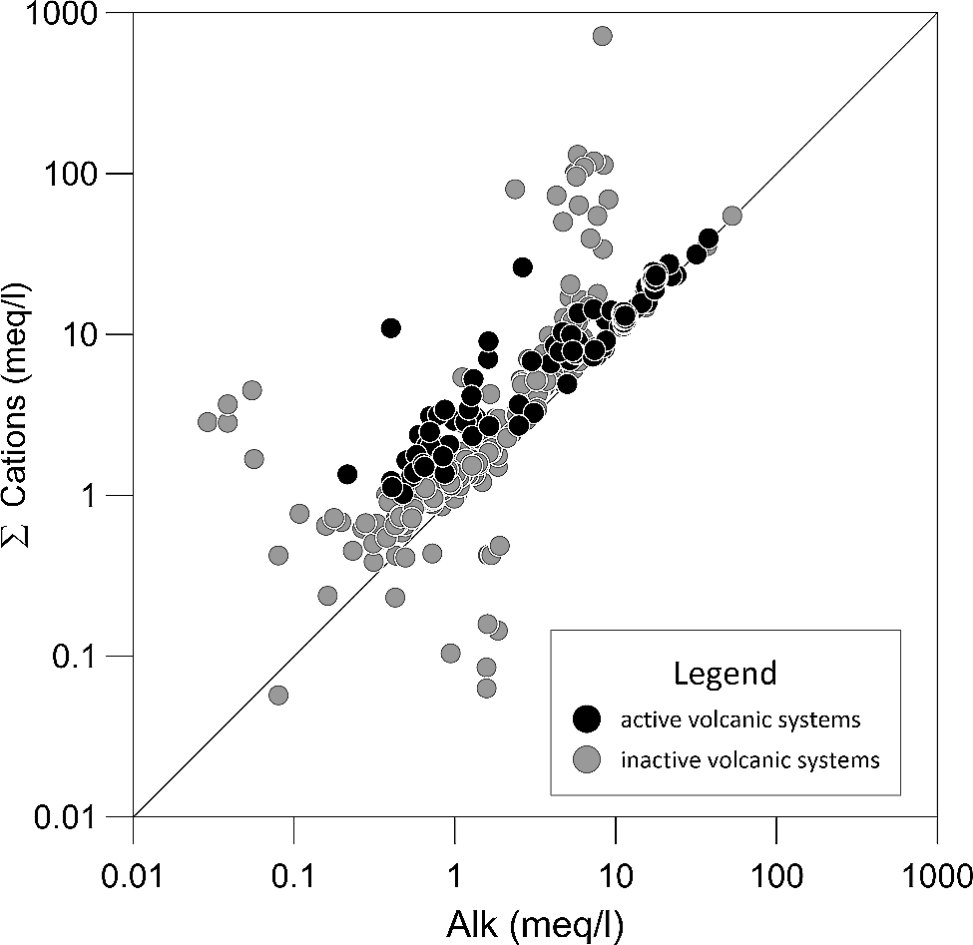
Sum of cations (Na, K, Ca, Mg) versus alkalinity for the groundwaters circulating in basaltic aquifers worldwide

Major elements concentration broadly shows a similar pattern in the aquifers worldwide. Taking into account the whole dataset, the sum of cation equivalents very often matches (or equals) the total alkalinity with most of data falling very close to the 1:1 line in Fig. 3. Even if the data reported in this work probably do not represent the totality of studies of water composition in basaltic aquifers (many papers being continuously published), we believe that the analysis of several basaltic aquifers in different locations and climate conditions is useful in order to understand the element mobility in aquifers. Na and Cl show the largest concentration range, varying within five orders of magnitude (0.001 to 100 mmol/l) (Fig. 1). High concentration values (between 10 and 100 mmol/l) are related with an example of basaltic aquifer in a coastal area [38], where saline brines influence the composition of water circulating in the basaltic aquifer; this is clearly evidenced with the excess of cations with respect to the alkalinity in (Fig. 3). The effect of sea-salt (i.e. aerosol) on the composition of waters circulating in basaltic aquifers can also be interpreted by looking in detail and elaborating the data. For instance, considering a basaltic aquifer close to the ocean area (i.e. Benedetti et al., 2003 Mt Cameroon), if the contribution of sea-salt is subtracted from the sampled water composition the data fall on the 1:1 line in Fig. 3, while the non-corrected data tend to the depart from 1:1 line. This highlights how water composition circulating in the aquifer is related to several factors that, depending on their extent, may be relevant. Therefore, the correct estimation of the charge balance is crucial in understanding the processes occurring in the aquifer. In some cases, we interpret the excess of cation versus low alkalinity values (Fig. 3) such as an incongruence in the charge balance of the data (not always attained); this corresponds for example to the lowest concentration in Na and Cl. Regarding other major elements (Mg, Ca, K) and the anion concentration, their distribution appears constant through the dataset considered. Among other minor elements reported in the dataset (Fig. 1), B, Li, Rb, Sr present a limited variability range within one or two orders of magnitude, while Al and Fe are usually in very low concentrations. Silica, aluminium and iron are the most abundant elements in basaltic rock; on the contrary, waters circulating in basaltic aquifers show low concentration. This is because such an elements tend to be removed from the basalts and rapidly incorporated in secondary phases [20]. In active and inactive volcanic systems, the presence of CO_2_ contributes to determine similar values for both alkalinity and for the sum of cations. Water of aquifers in active volcanic systems have both higher cation concentration and alkalinity (Fig. 3). Indeed, in the aquifer, high CO_2_ partial pressure values enhance the element mobility, causing high values for alkalinity and the sum of cations. Several authors studied the element mobility in groundwater of active volcanic systems [17, 19, 22], considering also the possible mobilization of toxic elements. Flaathen et al. [16], described the neutralization of CO_2_-rich water in an Iceland basaltic aquifer, reporting a toxic metal mobility, limited to the initial stage of basaltic-CO_2_-rich water interaction. In these environments, the precipitation of calcite plays a role in scavenging the released heavy metal. Precipitation of carbonate to form travertine deposits have been reported in Etna and Eyjafjallajökull volcanoes [34, 35] where waters are generally supersaturated with respect to calcite; these studies evidence that other minor elements are efficiently trapped in carbonated minerals. The amount of CO_2_ dissolved in the aquifer and the consequent variation on water pH are then pivotal to assess the fluid evolution during the CO_2_-water basalt interaction.

### Charge balance and volatile elements behaviour during dissolution

**Fig. 4:**
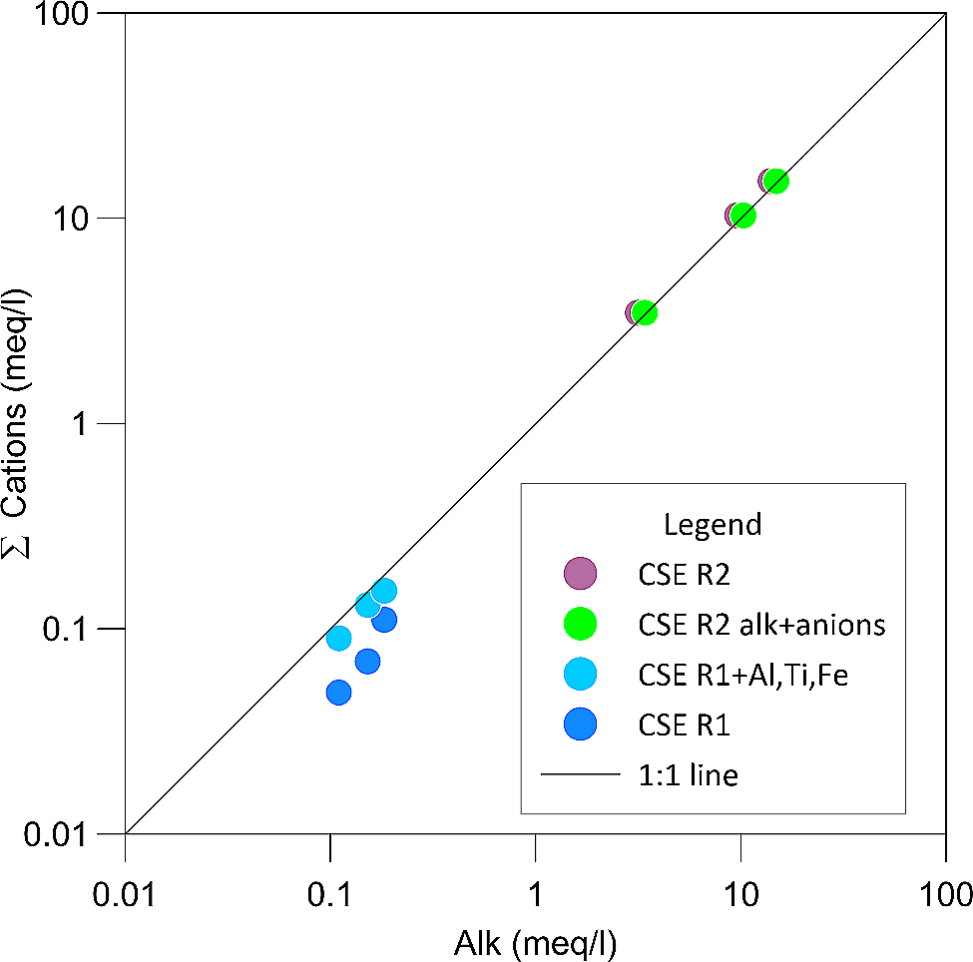
Sum of cations versus total alkalinity both expressed in meq/l. For the experimental set R1 cations, we show the sum of cations (Na, K, Ca, Mg) Fe, Ti, Al, Mn. For experimental set R2, we also plot the sum of cations versus alkalinity plus the anions determined in the water (F, Cl, S)

The charge balance is usually used to check the quality of analytical results, natural waters being electronically neutral. Indeed, if the sum of anion equivalents significantly differs from the sum of cation equivalents, the cause of the charge unbalance has to be searched in the missing determination of some element and/or in the analytical precision and accuracy. When the cation release is solely driven by the carbonic acid (formed by hydration of CO_2_), the total amount of dissolved cation equivalents should correspond perfectly to the sum of dissolved equivalent of ionic carbon species (namely HCO_3_^−^ and CO_3_^−−^). Considering our experimental results, we compared the sum of dissolved cation equivalents with the total alkalinity (Fig. 4) for R1 and R2 experimental sets. In R1 experiments, the sum of cations differs from the theoretical line with 1:1 ratio, while in the R2 set the results correlate perfectly, indicating that CO_2_ promotes the cation release. The difference from the theoretical line in R1 experiments may be attributed to the concentration of those elements considered in minor amounts. Indeed, if we consider, besides Na, Ca, Mg, K, the concentration of other elements such as Ti, Al, Mn and Fe, the sum of cation is higher, becoming very close to the alkalinity values. As regards the slight difference from the theoretical line with 1:1 ratio for the R2 experimental set, it indicates that also F^−^, Cl^−^, and SO_4_^2−^ have been released during the experiments. If we compare the sum of cation equivalents with the sum of total anion equivalents (i.e. Alkalinity plus F^−^, Cl^−^ and SO_4_^2−^), the result fits the theoretical line 1:1 perfectly, indicating that the charge balance was attained and also that the release of anions from the whole rock contributes to the charge balance. Such an effect was neglected by Liotta et al. [39] when discussing the CO_2_ driven weathering. However, here we highlight that the anions released from the whole rock contribute to about 6–8% of the total dissolved anion equivalents.

**Fig. 5:**
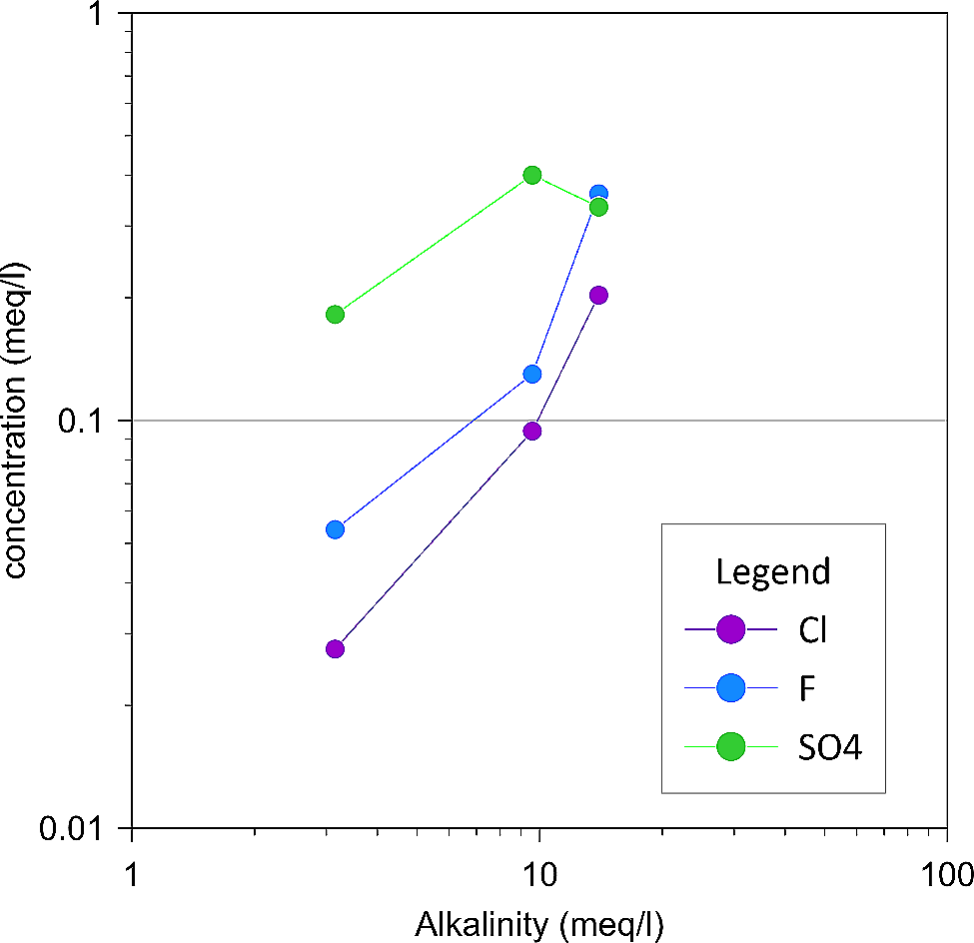
Alkalinity versus concentration of F, Cl and SO_4_ for the experimental set R2, in experimental set R1 these elements were below the detection limit

We analysed rock samples for their content of Cl, S, and F in order to evaluate the release of these elements during water rock interaction. The content of these elements in our experiment solutions R-1 were under the IC detection limit, while those from R-2 run (with different rock-water ratios) were well detected (within the calibration curve) and positively correlate with the total alkalinity (Fig. 5). This enabled assuming that CO_2_ driven weathering also allows the release of anions. Volatile elements should mainly be hosted in the glass matrix of the rock sample since basaltic rocks do not usually contain mineral phases with significant amounts of volatiles in their lattice. Therefore, their content in our experimental solutions testify to the dissolution of the glass matrix, which represents only a fraction of the total volume of the rock. Considering natural systems, for instance Etnean groundwater, the water composition is the result of several processes occurring in the aquifer such as the contributions of deep reservoir fluids and the volcanic plume. Magmatic CO_2_-rich fluids dissolving in groundwater, and the plume contribution in terms of acidic species and elements, are hence relevant in determining the composition of these waters. As suggested by Aiuppa et al. [17], water pH and Eh determine the direction of the evolving processes, and this is indicated by the good correlation between element concentrations, pH and alkalinity. However, other processes underlying the composition of groundwater should be taken into account and may be discussed in light of our experiments. These processes include a direct input through bulk deposition on the composition of infiltrating waters. Liotta et al. [39] observed that groundwaters at Mt. Etna are mainly affected by plume-derived weathering (PDW), CO_2_ -driven weathering (CDW), and plume-derived elements (PDE). In order to quantify the relative contribution of each process/source, Liotta et al. [39] performed a multiple regression with respect to Cl and alkalinity, being representative of PDW + PDE and CDW, respectively (see Liotta et al. [39] for details). They found that the sum of the cation equivalents was well described by the multiple regression, the coefficient of determination being r^2^ = 0.96 and the intercept about 3% of the sum. Na, Mg, K, and Ca show different coefficients of determination (0.93, 0.96, 0.78, 0.59, respectively) but high enough to indicate that PDW + PDE and CDW are the main sources of these elements. Liotta et al. [39] also sought to compute the relative contributions of PDW + PDE and CDW for each element. Such a computation is based on the values predicted by multiple regression and residuals indicate how far the observed values are from the predicted ones ([39] for details). However, our experimental results indicate that a significant amount of Cl is released during water-rock-CO_2_ interaction. In light of this, PDW + PDE fractions could be overestimated.

### Experimental data

**Fig. 6:**
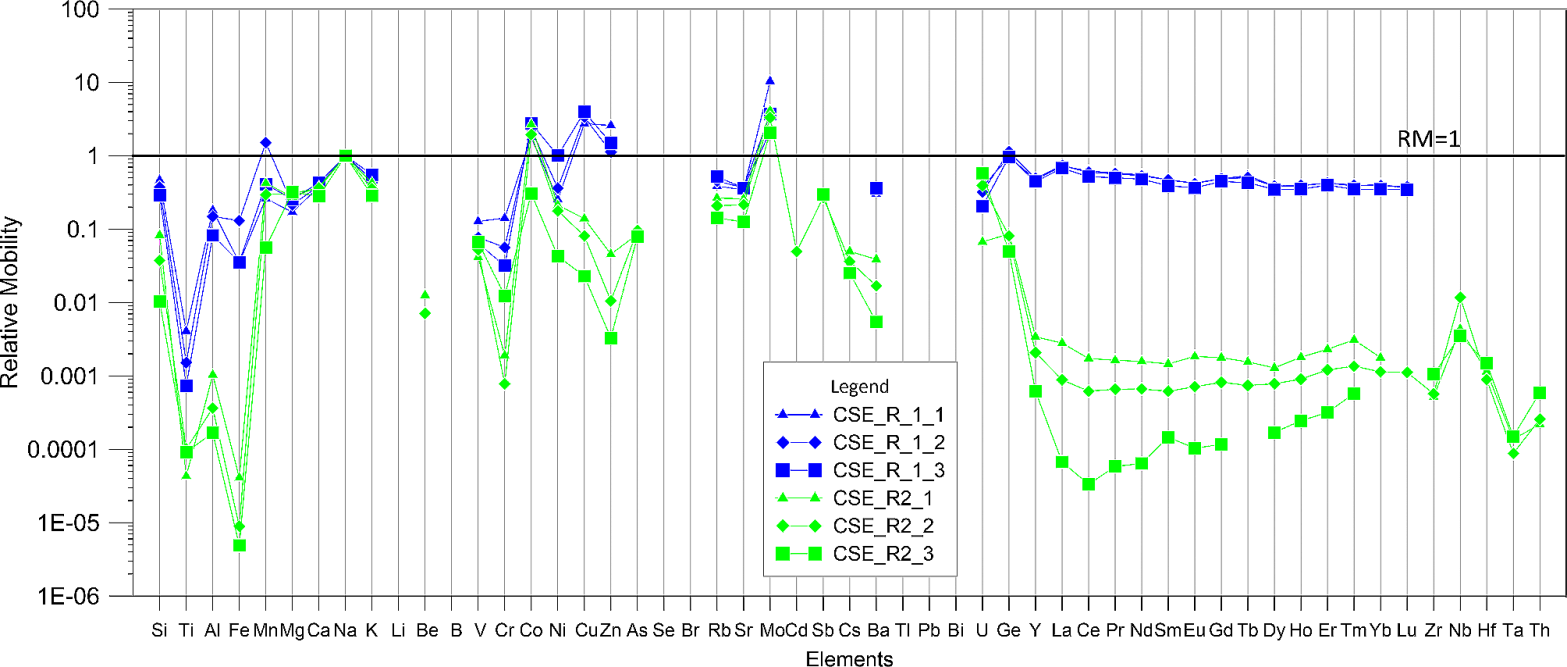
Relative mobility of major and trace elements in the solution after the water-CO_2_ basalt interaction experiments. The solid line refers to the relative mobility value of 1 to discriminate the element with relative mobility value major or minor of one

During rock-water interaction processes, the reaction kinetics and the reaction surfaces play a role in controlling the element mobility. In a first order of approximation, we consider the experimental conditions established in the two experimental sets as two different steps of the water interaction process occurring in natural systems. R1 and R2 experiments were performed using two different rock water ratios and each experimental set includes a progressive increase of this ratio. Following this rationale, we obtain two groups of water solutions that differ for pH values and alkalinity. Water solutions of experimental set R1 indeed always have pH < 5.5 and alkalinity lower than 0.2 meq/l, while in R2 experimental solutions pH is higher than 5.5 (and up to ∼ 7) and alkalinity ranges between 3 and 14 meq/l. Hence, we consider the experimental set R1 as the first step of the rock water-rock interaction process that intends to simulate the CO_2_-rich water interacting with a basaltic rock. In the second experimental set, the higher amount of rock powder simulates a situation in which a complete neutralization of the carbonic acid by the basaltic rock occurred. The rock-water ratio is changed in order to buffer the CO_2_-derived acidity of water. Since Etnean groundwater has pH values always higher than 5, we can assume that the R2 experimental set is more representative of natural conditions where micro fractures and pores occupied by water would be volumetrically subordinate to the total volume of the rock. Concentrations of major and trace elements show considerable differences in experimental sets R1 and R2, and major elements (Na, K, Ca, Mg, Si) including other minor elements such as Ti, Mn, Co, Ni, Rb, Sr, Mo are more abundant in R2 solutions and they correlate with the alkalinity. Other minor constituents such as Al and Fe display a more complex behaviour, being more abundant in R1 solutions with respect to R2. In terms of relative abundances, the major elements concentration in R1 is Si > Na > Ca > Al > Mg > K > Fe > Mn, while in the R2 dataset Na > Ca > Mg > Si > K > Mn > Fe > Al. The observed abundances do not concur with those of basaltic rocks (Si > Al > Fe > Ca > Mg > Na > K > Mn) since the weathering of basalt is not a congruent process and during the dissolution of igneous minerals, elements may be removed by the precipitation of secondary minerals. Although our experimental methodology and rationale may differ from those of previous experimental studies [9 and reference there in], we compared our results with both laboratory experiments and observations of natural systems. To this end, we used the experimental solutions and rock analyses to calculate the element relative mobility (RM):

$$RM{\text{ }} = {\text{ }}\left( {X/{X_r}} \right)w/\left( {X/{X_r}} \right)r$$

where X is a selected element, *w* and *r* refer respectively to the water (in our case to the experimental solution) and rock; X_r_ is the reference element. The quantification of relative mobility was applied on the study of rivers draining basaltic terrain [29, 40], as well as on the groundwater hosted in a volcanic edifice [17, 23, 29]. Gislason et al. [29] chosen sodium as the reference element given its strong chemical mobility during weathering and being the most mobile cation in southwest Icelandic waters. In order to compare our result with previous studies, we also used Na as a reference element. Relative mobility for major and trace elements for both experimental sets is shown in Fig. 6.

**Fig. 7:**
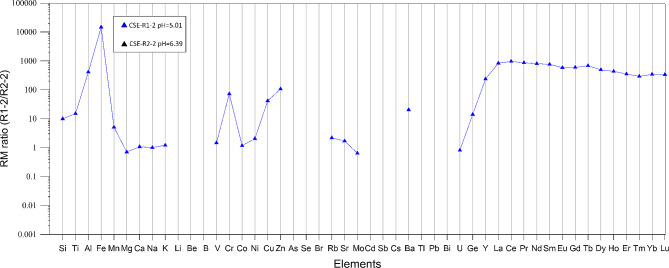
Relative mobility ratio calculated between the experimental set R1-2 and R-2-2. In the key, we reported the pH of the water solutions considered

Considering the experimental set R1, several elements have RM ≥ 1 such as Mn, Ni, Cu, Zn, Mo, Co, Ge, while in the experimental set R2 only Co and Mo have RM > 1. These latter elements have RM1 > 1 (except R2-3) regardless of the rock water ratio, evidencing its strong mobility during the weathering processes. [17] highlighted that Mo and other oxo-hydroxo anion forming elements are the most mobile elements in the Etnean aquifer, due to the tendency to form soluble oxo-hydroxo complex; this behaviour has also been observed in other basaltic aquifers such as Jeju island [23]. Arnórsson and Óskarsson [41] discussed the tendency of Mo to accumulate in water proportionally to the temperature and interaction time with the basaltic rock. As the main source of Mo, they authors consider plagioclase and pyroxene minerals as well as the volcanic glass. Due to the low crystallinity of the rock sample used in our study, we consider the dissolution of the glass matrix as the main source of Mo. All other elements determined in the experimental solutions have RM < 1 (except Mn in the R2-2) and values differ on the basis of the experimental solution considered (Fig. 6). Alkaline and alkaline-earth elements have similar RM values (1 < RM < 0.01) regardless of the rock-water ratio; only barium RM seems to vary with pH, which results higher in the experimental set R1 that has pH in the range 5.05–5.13. The RM of alkalis and alkaline earth elements in the R1 experimental set is Na > K > Ca > Mg and only in the experimental set R2-2 and R2-3 do we observe Na > K > Mg > Ca. The relative mobility of these elements is similar to that reported by Flaathen et al. [19] for Icelandic waters. It differs with respect to the relative mobility described by Aiuppa et al. [17] for Etnean groundwater, in which mobility is in the order Na > Mg > K > Rb > Ca > Cs > Li > Sr > Ba, similarly to Jeju island groundwaters [23]. In our experimental sets, the relative mobility is in the order of Rb > Sr > Cs > Ba and is usually lower than Ca and Mg. Previous studies related the mobility of Ca, Sr and Ba in basaltic aquifers with the presence of plagioclase and its abundances in the volcanic rocks. As discussed by Aiuppa et al. [17], the small ionic radius of Ca with respect to Ba explains the greater affinity of this latter element to the solid phase. The barium relative mobility shows a very high variability, being similar at low pH conditions to other alkaline earth elements, while at higher pH conditions it appears less mobile with relative mobility values in the range 0.01 and 0.1; in general, it shows an inverse correlation with alkalinity. Although to a lesser extent, at pH > 5 values (i.e. R2) also Sr and Rb show relative mobility values lower than those attained for pH < 5 (R1) and inversely correlate with alkalinity. In their study on Etnean groundwaters, Aiuppa et al. [17] discussed the high mobility of magnesium in waters as the result of the strong undersaturation in olivine and pyroxene caused by the low pH and temperature conditions during weathering processes. The trachy-basaltic rock used for our study presents a high weight% of plagioclase, as phenocrystal and microlite in the groundmass. We attribute to this the higher mobility of Ca with respect to Mg even if the differences in relative mobility values or absolute abundance appear generally low. During basalt weathering, silica, the most abundant element in basaltic rock, is mobilized together with other elements. Its abundance remains low in water compared to the amounts contained in basalts and with respect to the other major elements silica is not ionized but usually transported as multinuclear species [42]. It usually occurs as ortho-silicic acid and Si(OH)_4_ under neutral and slightly alkaline pH ranges, whereas under alkaline conditions the solubility of SiO_2_ is enhanced and forms other compounds. Several studies ([43, 44] and reference therein) discussed the formation of an amorphous silica layer by passivating the surface of minerals and glasses that together with the precipitation of secondary Si-bearing minerals cause a progressive decrease of the amount of silica in waters [8]. In our experimental sets, we observed that the mobility of silica is consistently higher in R1 irrespective of the rock-water ratio, while it decreases and correlates with the above-mentioned ratio in the experimental set R2. This trend is even more pronounced for Ti, Al, Fe, and other transitional metals such as Ni, Cu, and Zn. Relative mobility of Ti, Al, Fe decrease up to four orders of magnitude (in the case of Fe) from the experimental set R1 to R2 (Fig. 5) [8, 10]. using a different experimental setup, obtained low relative mobility values for Al, Fe and Ti, suggesting that this behaviour is common also in natural basaltic groundwaters. However, they showed that during the CO_2_ impulse (i.e. water pH < 6), the mobility of Al and Fe resulted much higher, similarly to our experimental results; this indicates that for pH ≤ 5 the mobility of Al and Fe is strongly enhanced. Except for V, Co and Mn, other transition metals such as Ni, Cr, Cu and Zn have greater mobility at low pH conditions. Transition metals have different oxidation states and their mobility is closely related with the formation of oxo-hydroxis complexes. Aiuppa et al. [17] showed that in Etnean groundwaters transition metals are from averagely mobile to immobile, with Al as the least mobile element. In the experiments of Galeczka et al. [8] during the input of CO_2_ in the system, manganese become the most mobile element while V and Ti are among the less mobile. In our experiments, we observe that vanadium and manganese RM values are similar for both experimental sets, while the relative mobility of Cr, Ni, Cu, and Zn vary up two orders of magnitude in relation to the water pH. We cannot exclude any influence of redox conditions variation during the experiments that can lead to the formation of soluble oxy-hydroxy compound, but in any case the water pH conditions at the first stage of rock-water interaction have a great influence on the mobility of these elements. In the experimental set R2, the increase in pH and alkalinity probably determined the formation of insoluble iron oxy-hydroxide compounds that scavenge other metals such Cr or Zn and Cu, as evidenced in several others studies [5, ]. In Fig. 5, rare earth elements (REE) show the most evident variation in RM values in relation to the experimental set considered, and hence in relation to the water pH and alkalinity. Rare earth elements have RM values in the range 0.1-1 in the experimental set R1, while in R2 we obtain RM values up to three orders of magnitude lower. During chemical weathering, rare earth elements (REEs) are released from primary minerals and then retained in the weathering products [31, 45, 46 and reference therein] such as clay minerals. REE may be complexed by a variety of ligands, for instance in alkaline water with CO_2_ partial pressures similar to the PCO_2_ of the atmosphere, REE are complexed by the formation of carbonate [47, 48]. In our experiments at low water pH conditions rare earth elements are strongly mobilized, and RM values are comparable with those of alkaline and alkaline earth elements. In the experimental set R2, alkalinity is up to two orders of magnitude higher with respect to the experimental set R1, which may have favoured the complexation of rare earth elements by carbonates and the consequent subtraction from the liquid phase. In Fig. 7, we show an enrichment ratio by dividing the relative mobility of the experiments R1-2 and R2-2. For some elements, the relative mobility increases from 100 to 10,000 times, the elements whose mobility depends on pH clearly deviate from 1. In detail, Fe is the most sensitive, followed by REEs, Al, Zn, Cr, Cu, Ba, Ge, Ti, Si and Mn.

### Implications for CO_2_ sequestration into mafic rocks

Rock water-CO_2_ interaction processes have several implications for carbon storage in natural systems. The CarbFix pilot project in Iceland [10] was the first large scale experiment aimed at investigating the potential of in-situ mineral carbon storage in basaltic rocks. It represented the convergence point for several theoretical studies and laboratory experiments focused on understanding the element mobility in waters, alteration of mineral phases and precipitation of secondary mineral phases [10, 49 and reference therein]. The injection of CO_2_-saturated waters enhances the basalt dissolution, leading to different degrees of element mobility. Galeczka et al. [9] during laboratory experiments, performed with a flow systems reactor, evidenced that the first hours of CO_2_-water basalt interaction are dominated by the rock dissolutions. In this phase, in which water pH may be lower than 6, the rapid mobilization and immediate precipitation of Si-Al bearing phases can occur. The high elements mobility in the first stages of the CO_2_-rich waters interacting with the mafic rocks also regards several metals, for instance Co, Cu, Zn and REEs among others. Our experimental results corroborate these aspects, highlighting that elements considered less mobile or immobile become mobile in the first stage of rock-water interaction. Gysi and Stefánsson, (2012a, b) [7, 50] through their experiments and numerical modelling, recorded the early precipitation of Fe-carbonates at elevated CO_2_ concentration and pH < 6.5. They also described a competing reaction path between clays and carbonates at low pH conditions, while at higher pH the competition is between zeolites, clays and carbonates, which controls the mobility of Ca, Mg and Fe. The rapid formation of secondary Al-Si bearing phases and Fe-carbonates causes the passivation of the primary rock that brings to a consequent slowing of the rock dissolution [43]. All these processes occurring at low pH conditions, together with the water-rock reaction surface, may control the entire process of mineral dissolution and mineral carbonation rates. As stated by Gysi and Stefánsson [50], low dissolution rates cause the early precipitation of secondary phases that leads towards a system lock-up with a consequent inefficiency in carbonate mineralization due to the unavailability of pore space and cations. Galeczka et al. [8] studied the mobilization of metals during water basalt interaction at high CO_2_ pressure. In their experiments, the CO_2_ pulses increased more than 100 times the mobility of some elements, among these Al, Fe, Cr, and Mn exceeded allowable drinking water limits (European Directive). Aquifers in basaltic volcanoes may represent a natural analogue in which meteoric water, groundwater and volcanic CO_2_ interact with mafic rocks. pH of natural waters in basaltic aquifer ranges between 6 and 8; these waters are clearly neutralized by the rocks, but on the contrary, meteoric water or volcanic CO_2_ saturated water may have lower pH values and favour the dissolution process. The release of metal during the intense weathering of the basaltic rock driven by the rising of magmatic CO_2_ may produce metal concentrations that exceed the admissible concentrations fixed by European Union of drinking waters as evidenced by Giammanco et al. [51] for Etnean groundwaters. In a natural system such as the Etna volcanic system, the high metal mobility is evidenced by the occurrence oxy-hydroxys deposits as in the case of San Giacomo valley, on the eastern flank of Etna mountain. There, a large thick of reddish mud characterizes the water emerging site; from a compositional point of view (supplementary material), this mud has an iron content in the range 56–58 wt% and SiO_2_ in the range 12–13 wt%. As also evidenced by our experiments, even if metals may be released during the first stages of water-CO_2_ rock interaction, they are then incorporated in the precipitation of carbonate and Fe oxy-hydroxides. In the complexity of natural systems, the precipitation of carbonate to form travertine deposits [34, 52] scavenges metals dissolved in waters and represents a natural mechanism of C storage within solid phases. Flaathen et al. [16] through numerical modelling, showed that in Icelandic groundwaters when pH exceeds 7.7 dolomite precipitates while precipitation of calcite occurs at pH ∼ 9, and the authors suggested a limited mobility of toxic elements. The study of groundwaters in active volcanic systems hence presents several analogies with the effects and the processes occurring when CO_2_-rich water is injected in basaltic aquifers.

## Conclusions

CO_2_-rich waters interacting with mafic rocks is a common process in active and inactive volcanic systems and in those sites where mafic rocks are identified as a potential reservoir for atmospheric CO_2_ storage. In this work, we first presented a review of groundwaters composition circulating in basaltic aquifers worldwide. This compilation of data highlighted the global correlating relationships between the alkalinity and the sum of cations of groundwaters circulating in basaltic aquifers when the cation release is solely driven by the carbonic acid (formed by hydration of CO_2_). The excess of cations over alkalinity may be interpreted as the result of other processes, for instance the contribution of brines or the effect of marine aerosol; hence, the correct estimation of the charge balance is crucial in understanding the processes occurring in the aquifer.

We performed two series (R1 and R2) of basalt-water-CO_2_ interaction experiments in order to gain further insights on the element mobility during the interaction processes. The most relevant results can be summarised as follows:

- the experimental sets differ for pH and alkalinity and intend to simulate two steps of the water-rock interaction process. As a consequence, major and trace elements show considerable differences between the experimental set R1 and R2. Na, K, Ca, Mg, Si including other minor elements such as Ti, Mn, Co, Ni, Rb, Sr, Mo, are more abundant in R2 solutions and they correlate with the alkalinity, whereas other minor constituents such as Al and Fe exhibit a more complex behaviour, being more abundant in R1 solutions with respect to R2.
- the relative abundances of elements in the experimental solutions do not concur with those of basaltic rocks since the weathering of basalt is not a congruent process and during the dissolution of igneous minerals, elements may be removed by the precipitation of secondary minerals. Considering the elements in terms of relative mobility, low pH conditions increase the mobility of some elements. This behaviour is particularly evident for Al and Fe as well as for rare earth elements, which at water pH ≤ 5 have relative mobility values three orders of magnitude higher with respect to the relative mobility of elements in the experiments with pH > 6.
- Basalt water-CO_2_ interaction processes have several implications for carbon storage in natural systems. The injection of CO_2_-saturated waters enhances the basalt dissolution, leading to different degrees of element mobility. Our experimental results highlight and corroborate the fact that at low pH conditions elements considered less mobile or immobile become mobile during the first stage of rock-water interaction. The release of metal during the intense weathering of the basaltic rock driven by the rising of magmatic CO_2_ may produce metal concentrations that exceed the admissible concentrations even if precipitation of secondary phases and carbonation may stabilize metals together with carbonates.

## Data Availability

Upon publication, data will be accessible.
